# Plant Biodiversity and Genetic Resources Matter!

**DOI:** 10.3390/plants9121706

**Published:** 2020-12-04

**Authors:** Andreas W. Ebert, Johannes M. M. Engels

**Affiliations:** 1 World Vegetable Center, 60 Yi-Min Liao, Shanhua, Tainan 74151, Taiwan; 2 Alliance of Bioversity International and CIAT, Maccarese, 00054 Rome, Italy; j.engels@cgiar.org

**Keywords:** genetic erosion, agrobiodiversity, crop wild relatives, ex situ and in situ conservation, plant breeding, diversification, climate change, sustainability, food and nutrition security, policy

## Abstract

Plant biodiversity is the foundation of our present-day food supply (including functional food and medicine) and offers humankind multiple other benefits in terms of ecosystem functions and resilience to climate change, as well as other perturbations. This Special Issue on ‘Plant Biodiversity and Genetic Resources’ comprises 32 papers covering a wide array of aspects from the definition and identification of hotspots of wild and domesticated plant biodiversity to the specifics of conservation of genetic resources of crop genepools, including breeding and research materials, landraces and crop wild relatives which collectively are the pillars of modern plant breeding, as well as of localized breeding efforts by farmers and farming communities. The integration of genomics and phenomics into germplasm and genebank management enhances the value of crop germplasm conserved ex situ, and is likely to increase its utilization in plant breeding, but presents major challenges for data management and the sharing of this information with potential users. Furthermore, also a better integration of in situ and ex situ conservation efforts will contribute to a more effective conservation and certainly to a more sustainable and efficient utilization. Other aspects such as policy, access and benefit-sharing that directly impact the use of plant biodiversity and genetic resources, as well as balanced nutrition and enhanced resilience of production systems that depend on their increased use, are also being treated. The editorial concludes with six key messages on plant biodiversity, genetic erosion, genetic resources and plant breeding, agricultural diversification, conservation of agrobiodiversity, and the evolving role and importance of genebanks.

## 1. Introduction

The foundation of the current global food supply is based on thousands of years of phenotypic selection of plant species and genotypes with favorable traits for cultivation and human nutrition by early farmers. This selection process eventually led to the domestication of crop species and the development of landraces that were well-adapted to local conditions. The rediscovery of Mendel’s laws of trait inheritance in 1900 and Darwin’s concept of natural selection provided the basis of modern plant breeding [1], giving rise to modern crop varieties. The development of high-yielding, uniform commercial crop cultivars since the early 1900s, especially for temperate crops, and later during the Green Revolution also for tropical and subtropical crops, led to global annual productivity gains of 1.0% for wheat, 0.8% for rice, 0.7% for maize, 0.6% for millets, and 0.5% for sorghum [2]. However, the wide dissemination of these high-yielding cultivars caused a drastic replacement and progressive elimination of the locally adapted landraces, in particular in tropical and subtropical countries. Moreover, the often-narrow genetic base of modern cultivars made them more vulnerable to the incidence of new races of diseases or insect pests. As such, breeders gradually started to explore landraces and especially crop wild relatives (CWR; the wild species from which modern crops derived and their close relatives) with useful traits, such as pathogen and insect pest resistance as well as tolerance to drought, salinity, and low or high temperature, in order to introgress the respective genes and/or quantitative trait loci (QTL) into modern crop varieties to make them more resilient.

It is crucial to conserve CWR and landraces if we wish to retain sufficient genetic diversity for current and future plant breeding programs [3]. This can be achieved through in situ conservation of CWR in their natural habitats or through the continuous use of landraces by farmers on their own farm, and thus maintaining the evolutionary processes that result in adaptation. However, due to the alarming rates of genetic erosion in nature, it is critically important to complement in situ conservation with ex situ conservation in genebanks—either as dried seeds with low seed moisture content (3–7%) at freezing temperatures (amenable for orthodox seeds) [4], or in field genebanks, through in vitro culture or cryopreservation (rapid freezing and storage at extremely low temperatures) [5]. The latter concerns crops that do not produce orthodox seeds with desiccation and low temperature tolerance (recalcitrant seeds; e.g. avocado), or do not produce seeds at all as is the case for bananas, or are generally clonally propagated, as seeds are not true-to-type as for instance potato [5]. It should be noted that ex situ approaches ‘freeze’ the genetic diversity, as no (or very limited) possibilities for adaptive evolutionary processes take place.

Production systems and the underpinning genetic resources, including crop wild relatives, which are (still) found on both cultivated and protected land, and especially in natural ecosystems such as forests, are severely threatened due to drastic land-use changes, over-exploitation of resources and human-made and natural disasters. Climate change is already significantly affecting the distribution of plants and associated species, their population sizes, and life cycles [6]. Currently, two in five plant species are estimated to be threatened with extinction [7]. The major threats to plants in situ, as assessed for the Red List of Threatened Species by the International Union for Conservation of Nature (IUCN), are as follows: agriculture and aquaculture (32.8%), biological resource use (21.1%), modification of natural systems (10.8%), residential and commercial developments (10.5%), invasive species, genes, and diseases (6.5%), pollution (5.4%), and climate change (4.1%). Yet, all genetic diversity is far from known to humankind, for instance only in 2019 close to 2000 new species of plants were discovered and described for the first time from Asia (36%), South America (34%), Africa (12%), Oceania (8%), Europe (5%), and North America (5%) [7]. Among those newly discovered species, there are also species of high potential relevance for agriculture, such as six species of *Allium*, described by scientists in Turkey, as well as wild relatives of cassava (*Manihot esculenta*), yams (*Dioscorea* spp.) and sweet potato (*Ipomoea* spp.) encountered in Brazil. If we are serious about slowing down the on-going genetic erosion and saving those species which are threatened or close to extinction, as well as those currently still being discovered by science, whose potential is still unknown, it is mandatory to conserve the genetic diversity found especially in biodiversity hotspots of wild and domesticated biodiversity [8]. This will benefit current and future generations.

The current over-reliance on a handful of major staple crops has inherent agronomic, ecological, nutritional, and economic risks, and is unsustainable in the long run. The wider cultivation of today’s underutilized minor crops [9] as well as intercropping [10] provide more options to build much needed heterogeneity into increasingly uniform cropping systems. This, in turn, will help to cushion the negative impact of climate change and to maintain and enhance evolutionary processes, efficiency and resilience of agroecosystems, as well as enhancing dietary diversity and combating malnutrition [11]. Efficient adaptation strategies for agriculture in a dynamically changing climate require, among other measures, effective and rational conservation and sustainable utilization of agricultural biodiversity, both in situ as well as in genebanks, and facilitated access to genetic resources of crops and their wild relatives by plant breeders and other users [12]. International access and exchange of genetic resources relies on the safe movement of germplasm across borders [13], with germplasm health requirements clearly differing from one country to another, and this germplasm movement raises a multitude of policy issues and concerns regarding access and benefit-sharing, ownership, intellectual property rights and patents imposed on plant genetic resources for food and agriculture (PGRFA) and breeding lines [14], as well as implications of transgenic crops for biodiversity and sustainable agriculture [15].

We encouraged contributions to this Special Issue on plant biodiversity and genetic resources in the form of original research and review papers with the aim to disseminate and promote knowledge on a wide array of aspects in this important field, which underpins sustainable agriculture and food and nutrition security in a globalized world, facing multiple challenges. This Special Issue comprises 32 papers covering a wide array of aspects from the definition and identification of hotspots of wild and domesticated plant biodiversity [8] to the specifics of the conservation of crop germplasm and their wild relatives in situ and ex situ, including germplasm and genebank management and genebank phenomics, to enhance the value and utilization of crop germplasm in breeding. The various modes of ex situ germplasm conservation in the form of orthodox seeds, as in vitro collections and/or in liquid nitrogen, have been described [5], and special attention has been drawn to the importance of seed physiology for the successful long-term conservation of orthodox seeds [4]. One paper assessed the specific case of seed germination after 30 years of storage in permafrost [16].

Attention has also been drawn to the challenges of some crops and crop wild relatives that present specific hurdles for successful, long-term ex situ conservation, such as forage germplasm (grasses, herbaceous legumes, and fodder trees) [17], wild bananas presenting varying levels of seed desiccation tolerance [18], wild food plants [19,20], and crop wild relatives [3]. As crop wild relatives are mostly found in the wild and are rather difficult to be conserved ex situ, the merits of payment for ecosystem services for in situ conservation have been analyzed by Tyak et al. [21] for these germplasm resources, which are increasingly coming into the focus of plant breeders, given their broad genetic diversity, harboring many (new) traits of interest to build resistance to diseases and insect pests as well as tolerance to abiotic stresses into resilient modern crop cultivars. With respect to germplasm management, an example of the use of single nucleotide polymorphisms (SNP) markers for the identification of duplicate accessions of *Brassica oleracea* in genebank collections has been presented [22], while another paper highlights the importance of information management to assist in germplasm and genebank management and to enhance the use of germplasm [23]. Genebank phenomics is a rather novel approach in modern genebanking, and Nguyen and Norton [24] shed light on digital phenotyping methods that enable capturing traits during annual seed regeneration events to enrich genebank phenotypic datasets, thus adding value to crop collections and increasing their usefulness for the identification of traits of interest for breeding.

The international germplasm collections hosted by 11 CGIAR Centers include over 760,000 accessions of crops, forages, and trees [14], and constitute a major proportion of the international germplasm exchange. Over the last ten years, the CGIAR genebanks distributed over 1.1 million PGRFA samples to recipients in 163 countries. Therefore, the CGIAR Germplasm Health Units play a major role in safe global germplasm movement and exchange and the prevention of the trans-boundary spread of pests and diseases [13]. Halewood et al. [14] elucidated the state of international and national access and benefit-sharing laws, noting some unresolved tensions regarding access and benefit sharing and digital genomic sequence information that have the potential to undermine international cooperation to conserve, share and use germplasm for use in food and agriculture.

Several papers highlight the importance of vegetables as well as traditional, underutilized and wild food plants for food and nutrition security in general [19,25], in pilot studies in Kenya [20], and on atolls in the South Pacific [26], including specific crops, such as Hairy Stork’s Bill (*Erodium crassifolium*) [27], and the sister of the common pomegranate (*Punica protopunica*), the latter also having interesting medicinal properties [28]. A number of papers focus on the genetic diversity of specific crops or specific traits in a range of food crops for the benefit of plant breeding, such as genome-wide association mapping for stripe rust resistance in spring wheat [29], diversity studies for drought and heat stress in maize landraces [30], nitrogen fixation and water use efficiency in common bean landraces and cultivars in Honduras [31], species identification of Katsouni pea on Greek Islands [32], wild potato germplasm evaluation for starch content and nitrogen utilization efficiency [33], diversity, population structure and marker-trait association for 100-seed weight in a safflower (*Carthamus tinctorius*) germplasm panel [34], the composition of Cypriot grapevine varieties [35], species assignment, genetic diversity and phylogeographic relationships of wild germplasm of macadamia [36], genetic diversity and population structure of *Rhododendron rex* subsp. *rex* [37], and genetic distinctiveness of a Sicilian manna ash (*Fraxinus angustifolia*) collection [38]. A further paper looks at the genetic distinctiveness of cogongrass (*Imperata cylindrica*), an invasive species in the Southern United States [39].

Rosero et al. [40] advocate a dual strategy of breeding for drought tolerance in staple crops and introducing drought-tolerant, underutilized species in cropping systems to enhance their resilience to drought. In the context of public breeding programs in 18 developing countries, Galluzzi et al. [41] analyze the specific role of genetic resources in breeding for climate change. The importance of vegetable genetic resources for nutrition security and vegetable breeding is highlighted in a paper by Ebert [25].

## 2. Key Messages

### 2.1. Plant Biodiversity

Plant biodiversity encompasses the ecosystem level, species diversity, and the genetic diversity within species. While biodiversity hotspots are mainly defined by species richness, agrobiodiversity hotspots refer to the centers of origin and diversity of (major) crops and their wild relatives, harboring genetic resources that are of high and increasing relevance for plant breeding. Pironon et al. [8] observed spatial congruence between biodiversity and agrobiodiversity hotspots and proposed a unifying concept, taking into consideration not only species richness but also the multiple benefits plants offer to humankind in terms of ecosystem and agro-ecosystem functions, the inter- and intra-specific diversity of the chemical properties of plants for human nutrition and medicinal use, as well as the provision of gene sources for plant breeding. Rather than considering only major global food crops, agrobiodiversity conservation and use strategies should explicitly include the huge number of edible plants, which are currently underutilized and/or only of regional or often local importance, including wild food plants harvested in the wild that provide significant food and other benefits to humanity [19,20].

### 2.2. Genetic Erosion

As noted by Antonelli et al. [7], two out of five plant species are estimated to be threatened with extinction. The major threats to plants are of anthropogenic nature and encompass agriculture, overexploitation of biological resources in the wild, modification, fragmentation, and destruction of natural ecosystems, rapidly expanding residential and commercial developments, pollution, and climate change. Van de Wouw et al. [42] highlighted three major evolutionary bottlenecks which led to an enormous reduction in the genetic diversity of cultivated plants. A first major drop in genetic diversity occurred during the development steps from the wild ancestors into the domesticated forms (domestication bottleneck), followed by a dispersal bottleneck when only a small population or even a single plant may have been the foundation of the introduced crop, far away from the area of domestication (e.g., introduction of soybean to North America and the introduction of coffee, traceable to one single plant, to South America). Finally, the modernization bottleneck is due to the success of modern plant breeding and the wide dissemination and adoption of high-yielding cultivars that led and continue to lead to a drastic replacement of landraces. If we are not trying to halt the ongoing processes of dramatic genetic erosion and habitat destruction and fragmentation, we are threatening the web that is sustaining our lives.

Several papers in this Special Issue have addressed genetic erosion aspects and provided suggestions and experiences regarding how this erosion can be stemmed for wild and cultivated biodiversity [8], CWR [3,21], wild food plants [19,20], vegetables [25], and forages [17].

### 2.3. Genetic Resources and Plant Breeding

It is crucial to conserve landraces and CWR of crops if we wish to retain sufficient genetic diversity for current and future plant breeding programs, and to develop resilient cultivars that can withstand the multiple biotic and abiotic challenges that are exacerbated by climate change [3,25]. However, public sector breeders in developing countries are still confronted with obstacles such as accessing germplasm across national borders and the lack of appropriate technologies and skills to exploit sets of germplasm accessions composed of landraces and CWR [41]. It has been proposed that the commonly used estimated breeding value (EBV) of a parent in a cross could be expanded from individuals to species and populations, and in this case go beyond mere heritability values to additionally include crossing considerations (e.g., ploidy, mating system), evolutionary factors (e.g., phylogenetic relationship), and ecological factors (e.g., environment the species is thriving in) [8]. This multi-pronged approach of an expanded EBV could be used to produce a ranking of species for an individual breeding program, based on the traits required for a desired phenotype. Employing this EBV evaluation tool for characterizing the utility variation across species (and populations) could help define priority areas for in situ and ex situ conservation according to specific breeding targets.

Examples of conservation activities that impact on the use of the genetic resources that have been covered in this Special Issue include the following: easy access to genetic resources increases the capacity of breeders to respond to climate change and the availability of appropriate technologies [41]; access to traditional knowledge on the use of wild plant species [27]; a systematic association-mapping of wheat varieties with SNP markers was successfully used to associate adult plant stripe rust resistance with specific rust races, and results can be used in marker-assisted selection [29]; the analysis of a local genetic panel of manna ash with a continental dataset allowed conclusions on the presence of a possible glacial refuge, and thus facilitates the collecting and use of more genetic diversity [38]; the systematic characterization of ancient grape germplasm in Cyprus allowed the discovery of so far unnoticed genetic diversity [35]; literature searches and conducting field surveys allowed the identification of unknown wild food plants in Kenya [20]; fact sheets promoted the use of traditional food plants in the South Pacific [26]; the exploitation of the local genetic diversity of traditional pea landraces in Greece is fundamental for conservation practices and crop improvement through breeding strategies [32]; the evaluation of maize landrace accessions under heat and drought stresses resulted in invaluable sources of genes/alleles for adaptation breeding [30]; the review of recent efforts that build evidence of the importance of wild food plants in selected countries, while providing examples of cross-sectoral cooperation and multi-stakeholder approaches, contributes to enhancing their sustainable use [19]; the advances in conventional and molecular breeding for the drought tolerance of conventional staple crops, and the introduction of drought-tolerant neglected and underutilized species into existing production systems has the potential to enhance the resilience of agricultural production under conditions of water scarcity [40]; the utilization of advanced phenotyping tools, coupled with high-throughput genotyping, will accelerate the use of genetic resources and fast-track the development of more resilient food crops for the future [24]; and genomics-assisted breeding is increasingly facilitating the introgression of favorable genes and quantitative trait loci from wild species into cultigens, and will lead to a wider use of crop wild relatives in the development of resilient cultivars [25].

### 2.4. Agricultural Diversification

Relying on only a handful crops and on the enormous advances in plant breeding to feed the global population has greatly improved (global) food security, but has also contributed to malnutrition and has left farmers vulnerable to climate change. A meta-analysis of over 5000 original studies led Tamburini et al. [43] to the conclusion that agricultural “diversification enhances biodiversity, pollination, pest control, nutrient cycling, soil fertility, and water regulation without compromising crop yields”. Multispecies cropping systems constitute practical applications of ecological principles based on biodiversity, plant interactions, and other natural regulation mechanisms [44], and may lead to higher productivity, yield stability, ecological sustainability, and resilience to disruptions caused by climate change and other natural events. Diversification with a wider use of neglected and undervalued fruit and vegetable crops and semi-domesticated species, either intercropped with main staples in cereal-based systems or as stand-alone crops, would also contribute to the increased availability (and consumption) of nutritious food and, thus, help combat the triple burden of malnutrition [25]. However, as underutilized crops and semi-wild plant species rarely draw the attention of plant breeders, they often do not meet market standards and the expectation of consumers, and thus might slowly disappear with the advances in the breeding of the major food crops. Therefore, special attention should be given to the conservation and research of underutilized, threatened crop species and their associated genetic diversity by genebank curators. In addition, breeding efforts in these minor crops should be promoted, so that they will keep their place in farms, contributing to more sustainable production systems and benefitting consumers with diverse, often nutrient-dense food.

### 2.5. Conservation of Agrobiodiversity

The choice of the conservation approach, i.e., in situ and ex situ, as well as specific methods of the latter, will depend on the purpose of the conservation. In case we would like to maintain evolutionary processes that allow targeted genetic resources to evolve, we have to opt for in situ approaches. In cases where farmers are still actively depending on and managing crop diversity for their production, on-farm conservation would apply. Where long-term conservation is important, combined with easy access to diversity, genebank and other ex situ approaches are preferred.

About 80% of plant species produce viable seeds that are desiccation-tolerant and can easily withstand long-term low temperature storage; this presents the most convenient form of ex situ storage of germplasm of most domesticated crops and their close wild relatives. Special attention has been paid to the importance of seed physiology for the successful long-term conservation of orthodox seeds [4]. For other crops that do not produce orthodox seeds with desiccation and low temperature tolerance, or do not produce seeds at all, or are generally clonally propagated as seeds are not true-to-type, different conservation options are available. Those crops can be safely conserved in field genebanks, through in vitro culture or cryopreservation [5]. Crop wild relatives of the secondary and tertiary genepool are mostly found in the wild and are rather difficult to conserve ex situ. These resources are best maintained in situ, in their natural habitat, usually in protected and undisturbed areas where they can continue to evolve [3]. Payment for ecosystem services may be a useful tool to support the in situ conservation of CWR [21].

The availability of comprehensive information on the composition of genebank collections is an important prerequisite to further develop such collections and thus, to increase their utility for breeders and other users [23]. The on-going and increasingly accelerating genetic erosion, as well as the fact that a significant amount of ‘new’ genetic diversity is still being discovered every year, highlight the importance and urgency of conserving and systematically studying the genetic diversity found, especially in biodiversity hotspots of wild and domesticated biodiversity [8]. This will help in achieving the food and nutrition security of a still growing global population, and will secure other benefits provided by the plant kingdom, such as environmental services and the functional ingredients of plants.

### 2.6. The Evolving Role and Importance of Genebanks

Genetic resources conserved in genebanks are often considered to be sub-utilized for breeding purposes. This is at least partly due to the fact that most genebanks can offer users only limited, often incomplete passport and basic characterization data, while evaluation data are mostly missing. Characterization data are generally obtained during germplasm regeneration events and mostly focus on morphological descriptors with a high degree of heritability. The limited availability of genotypic and phenotypic information on the majority of genebank accessions is a major reason why breeders are hesitant to use genebank accessions in their breeding programs. A strategic three-step approach has been proposed by McCouch et al. [45] to enhance the mining of genetic resources conserved in genebanks. Such an approach combines genomics and phenomics with efficient information management to enhance the value of available germplasm, thus making it user-friendly for breeders. Enormous progress has been made on the molecular front with the availability of modern cost-effective high throughput molecular tools and advanced statistical analysis that enables the genomic selection of traits of interest. Although many QTL have been identified for biotic and abiotic stresses, their practical use is still limited due to the lack of high throughput phenotyping and molecular platforms [46]. Nguyen and Norton [24] suggested the use of digital high-throughput phenotyping methods that can capture traits of interest to breeders during annual seed regeneration events to enrich phenotypic datasets of genebank accessions. However, high initial investment costs, data capture and standardization, quality assurance, and data analysis are major technical challenges related to genebank phenotyping that restrict the number of genebanks which can adopt and effectively run a high-throughput phenotyping platform.

Finally, to make comprehensive information on germplasm accessions available for global users, there is a clear need for a cooperative platform for data collection, analysis and sharing. The International Plant Phenotyping Network brings plant phenotyping centers together and facilitates the sharing of technical advances in high-throughput phenotyping equipment, data capture and standardization for a range of crop phenotypes [47]. In order to facilitate the use of conserved germplasm, genebanks should consider offering this material in a suitable form to potential users. Examples of facilitating the use of germplasm are as follows: the splitting of heterogeneous accessions into uniform lines [48]; the generation of core, minicore [49] and trait-specific collections [50], as well as single seed descents [51]. In addition, the conservation and distribution of genetic stocks to breeders might facilitate the use of genetic resources and thus increase the availability of more genetic diversity by breeders. For an overview of the complex set of operations in a typical seed genebank, please see Hay and Sershen [52] (p. 4, Figure 1).

As reported by Halewood et al. [14], there is widespread concern, especially among developing countries, regarding the governance of digital genomic sequence information, leading to international disagreement over access and benefit sharing regulations. However, despite the enormous enthusiasm regarding the importance of dematerialized digital genomic sequences for the potential engineering of new plants, the conservation of accessions in physical form will remain important as genotyping needs the complement of phenotyping to be of relevance for plant breeders. Future research may show the value of physical collections in ways that no one can anticipate today [53].
